# Analyses of mitochondrial amino acid sequence datasets support the proposal that specimens of *Hypodontus macropi* from three species of macropodid hosts represent distinct species

**DOI:** 10.1186/1471-2148-13-259

**Published:** 2013-11-21

**Authors:** Abdul Jabbar, Ian Beveridge, Namitha Mohandas, Neil B Chilton, D Timothy J Littlewood, Aaron R Jex, Robin B Gasser

**Affiliations:** 1 Faculty of Veterinary Science, The University of Melbourne, Parkville, Melbourne, Victoria 3010, Australia; 2 Department of Biology, University of Saskatchewan, 112 Science Place, Saskatoon, Saskatchewan S7N 5E2, Canada; 3 Department of Life Sciences, The Natural History Museum, Cromwell Road, London SW7 5BD, UK

**Keywords:** *Hypodontus macropi*, Mitochondrial genome, Kangaroo, Wallaby, Sliding window analysis, Next-generation sequencing, Genetics, Systematics

## Abstract

**Background:**

*Hypodontus macropi* is a common intestinal nematode of a range of kangaroos and wallabies (macropodid marsupials). Based on previous multilocus enzyme electrophoresis (MEE) and nuclear ribosomal DNA sequence data sets, *H. macropi* has been proposed to be complex of species. To test this proposal using independent molecular data, we sequenced the whole mitochondrial (mt) genomes of individuals of *H. macropi* from three different species of hosts (*Macropus robustus robustus, Thylogale billardierii* and *Macropus* [*Wallabia*] *bicolor*) as well as that of *Macropicola ocydromi* (a related nematode), and undertook a comparative analysis of the amino acid sequence datasets derived from these genomes.

**Results:**

The mt genomes sequenced by next-generation (454) technology from *H. macropi* from the three host species varied from 13,634 bp to 13,699 bp in size. Pairwise comparisons of the amino acid sequences predicted from these three mt genomes revealed differences of 5.8% to 18%. Phylogenetic analysis of the amino acid sequence data sets using Bayesian Inference (BI) showed that *H. macropi* from the three different host species formed distinct, well-supported clades. In addition, sliding window analysis of the mt genomes defined variable regions for future population genetic studies of *H. macropi* in different macropodid hosts and geographical regions around Australia.

**Conclusions:**

The present analyses of inferred mt protein sequence datasets clearly supported the hypothesis that *H. macropi* from *M. robustus robustus, M. bicolor* and *T. billardierii* represent distinct species.

## Background

*Hypodontus macropi sensu lato* (Strongyloidea) is a nematode that occurs in the terminal ileum, caecum or colon of a range of macropodid marsupials (kangaroos and wallabies) and was first described as a hookworm-like nematode [1,2]. Various studies of *H. macropi* from hosts across vast geographical distances in Australia [3-7] did not reveal any significant morphological variation in the nematode among different host species, but particular predilection sites in the gut as well as host affiliations suggested that *H. macropi* represented a complex of morphologically indistinguishable, but genetically distinct species or groups which have a relatively high level of host specificity. To test this proposal, Chilton et al. [8] conducted a multilocus enzyme electrophoretic (MEE) analysis and subsequently [9] determined genetic variation in the second internal transcribed spacer (ITS-2) of nuclear ribosomal DNA (rDNA), and provided evidence of genetic differences in *H. macropi*.

Using single-strand conformation polymorphism (SSCP) and selective sequence analysis of the ITS-2, genetic variation in the nematode was found [10], with nucleotide sequence variability mainly in loops or bulges of the predicted secondary structure. Moreover, in the most extensive investigation of *H. macropi* to date [11], a molecular-phylogenetic analysis of 547 specimens representing all ten known macropidid host species of this nematode from across the Australian continent revealed clear genetic clades in each of *Macropus agilis*, *M. dorsalis*, *M. rufogriseus*, *M. bicolor*, *Petrogale persephone*, *Thylogale billardierii* and *T. stigmatica*. Another clade was represented by all specimens from *M. robustus robustus* and *M. rufus*, together with two examples of host switching by the nematode into *M. fuliginosus*.

These findings showed that *H. macropi*, as currently defined morphologically, might represent as many as ten cryptic species. However, to date, DNA sequencing studies have been conducted exclusively using the second internal transcribed spacer (ITS-2) of nuclear ribosomal DNA, which represents species prospecting (as defined in [12]), rather than clear evidence for the existence of separate species. Although ITS-2 has consistently provided specific identification of strongyloid nematodes [13-15], there is a need to provide independent evidence of genetic identity and differentiation. Various studies have shown that mt genomes provide barcodes for organisms [16-21]. Although nucleotide variation within species of nematodes is relatively high for the mt genes studied to date [18] and, thus, is not useful for specific identification, this is not the case for mt protein sequences. Indeed, amino acid sequence variation within species of nematodes examined to date is usually very low (0–1.3%) [17-19]. Therefore, amino acid sequences derived from the mt genome provide barcodes for species and for studying the systematics (taxonomy and phylogeny) of nematodes [20]. Importantly, phylogenetic analysis of mt amino acid datasets usually provides strong statistical support for the relationships of nematodes, which is not achieved using data from short sequence tracts.

In the present study, we sequenced the complete mt genomes of individuals of *H. macropi*, proposed to be distinct cryptic species based on MEE and ITS-2 data sets [8-11] from three different species of macropodid hosts. We undertook a comparative analysis of the amino acid sequence datasets derived from these genomes and conducted a phylogenetic analysis of these datasets using homologous datasets for other strongyloid nematodes for comparison.

## Results

### Characteristics and comparisons of mt genomes

The three mt genome sequences of three operational taxonomic units (OTUs) OTU-C, OTU-G and OTU-J of *H. macropi* from *M. r. robustus*, *M. bicolor* and *T. billardierii* and of *Macropicola* (*Ma*.) *ocydromi* (outgroup), were assembled from 73,189, 29,997, 20,3341 and 127,146 individual sequence reads, respectively. For individual OTUs, the number of contigs resulting from each assembly ranged from 69 to 508, with the longest and shortest contigs being 11,170 and 41 bp in length.

**Figure 1:**
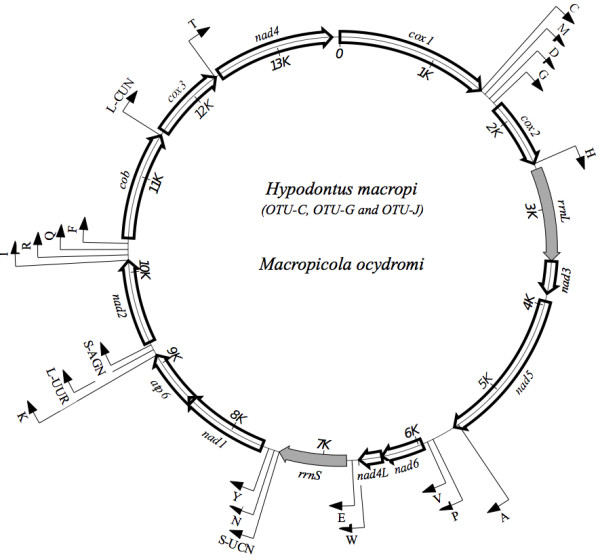
**Schematic representation of the circular mitochondrial genome of *Hypodontus macropi *and *Macropicola ocydromi.*** Three operational taxonomic units (OTUs) of *H. macropi* were from three different hosts (i.e., OTU-C from *Macropus robustus robustus*; OTU-G from *Macropus bicolor*; OTU-J from *Thylogale billardierii*) and *M. ocydromi* was from *Macropus fuliginosus.* Transfer RNA genes are designated using one-letter amino acid codes. Ribosomal genes are shaded.

The three circular mt genome sequences of OTU-C, OTU-G and OTU-J of *H. macropi* were 13699 bp, 13655 bp and 13634 bp in length, respectively (Figure 1). The mt genome of a closely related nematode, (*Ma. ocydromi*, was 13517 bp in length (Figure 1). Consistent with all species of related nematodes sequenced to date [18,20,21], the mt genomes of all three *H. macropi* OTUs and *Ma. ocydromi* contained 12 protein coding genes [adenosine triphosphatase subunit 6 (*atp*6), cytochrome *b* subunit (*cyt*b), cytochrome *c* subunits 1–3 (*cox*1-3), and the nicotinamide dehydrogenase subunits 1–6 and 4 l (*nad*1-6 and 4 l) genes], two ribosomal subunits [large (*rrn*L) and small (*rrn*S) subunit] and 22 transfer RNA (tRNA) genes (including two leucine and two serine tRNA genes). The overall mt genome structure for all OTUs of *H. macropi* and *Ma. ocydromi* was consistent with the gene arrangement GA2 (arrangement of mt genes) representing all other nematodes of the order Strongylida investigated to date [18,20]. The positions, lengths and start/stop codons of individual genes as well as amino acid sequence lengths of predicted proteins of the three OTUs are shown in Table 1. As expected for other strongyloid nematodes [18], no *atp*8 subunit is present, and all genes are transcribed from the forward strand; all protein coding genes had open reading frames (ORFs). For all three OTUs, the lengths of the protein-coding genes were in the following order: *nad*5 > *cox*1 > *nad*4 > *cyt*b > *nad*1 > *nad*2 > *cox*3 > *cox*2 > *atp*6 > *nad*6 > *nad*3 > *nad*4L (Table 1). The longest gene was *nad*5, and the amino acid lengths of *cox*1, *nad*4L and *cox*3 were the same for all OTUs (Table 1). The predicted nucleotide and amino acid sequences of each of the 12 protein-coding genes of *H. macropi* and *Ma. ocydromi* were compared. The mt genes of these OTUs had ATT, ATA and ATG as initiation codons; except for ATC as its initiation codon for *atp*6. All genes had complete termination codons, i.e., TAA and TAG, in contrast to some nematodes, such as *Ascaris suum*, *Necator americanus* and *Trichinella spiralis*, which have abbreviated stop codons (e.g., TA or T) for some genes [22-24].

**Table 1:** **Summary of mitochondrial genomes of *Hypodontus macropi *** **from three different hosts and *Macropicola ocydromi***

**Gene**	**Positions and nt sequence lengths (bp)**				**Initiation/Termination codons and amino acid sequence lengths**			
	**OTU-C**^**1**^	**OTU-G**^**2**^	**OTU-J**^**3**^	***M. ocydromi***	**OTU-C**	**OTU-G**	**OTU-J**	***M. ocydromi***
*cox*1	7-1576	7-1576	7-1576	7-1576	ATA/TAA (523)	ATA/TAA (523)	ATA/TAA (523)	ATG/TAA (523)
*trn*C	1581-1634	1583-1639	1580-1635	1578-1633				
*trn*M	1636-1695	1639-1698	1636-1693	1633-1692				
*trn*D	1698-1757	1704-1763	1694-1752	1695-1752				
*trn*G	1762-1819	1765-1822	1754-1810	1753-1808				
*cox*2	1819-2512	1822-2515	1802-2504	1808-2501	ATT/TAA (231)	ATA/TAA (231)	ATA/TAA (234)	ATT/TAG (231)
*trn*H	2514-2571	2518-2573	2507-2562	2506-2561				
*rrn*L	2573-3532	2578-3537	2565-3524	2568-3527				
*nad*3	3538-3871	3542-3875	3521-3854	3528-3864	ATA/TAG (111)	ATT/TAG (111)	ATG/TAA (111)	TTG/TAA (112)
*nad*5	3974-5534	3979-5539	3961-5521	3972-5532	ATG/T (536)	ATG/T (536)	ATG/T (534)	ATG/T (536)
*trn*A	5535-5591	5540-5596	5522-5578	5533-5589				
*trn*P	5837-5893	5841-5899	5828-5882	5818-5874				
*trn*V	5894-5950	5905-5961	5884-5939	5875-5930				
*nad*6	5950-6382	5961-6393	5940-6372	5936-6362	ATT/TAG (144)	ATT/TAG (144)	ATT/TAA (144)	ATA/TAA (142)
*nad*4L	6386-6617	6397-6628	6378-6609	6364-6595	ATT/TAG (77)	ATT/TAA (77)	ATT/TAG (77)	ATT/TAG (77)
*trn*W	6627-6684	6638-6696	6613-6669	6603-6660				
*trn*E	6689-6745	6705-6761	6671-6727	6660-6716				
*rrn*S	6741-7441	6760-7460	6730-7430	6709-7409				
*trn*S (UCN)	7442-7498	7460-7514	7418-7482	7395-7464				
*trn*N	7497-7554	7513-7570	7482-7538	7462-7518				
*trn*Y	7563-7617	7573-7629	7544-7599	7522-7576				
*nad*1	7690-8527	7646-8498	7619-8471	7593-8445	ATG/TAG (279)	ATA/TAA (284)	ATA/TAG (284)	ATG/TAG (284)
*atp*6	8536-9133	8501-9098	8486-9080	8473-9070	ATT/TAG (200)	ATT/TAA (200)	ATC/TAG (198)	ATT/TAA (200)
*trn*K	9135-9196	9101-9163	9084-9145	9076-9138				
*trn*L (UUR)	9200-9254	9165-9220	9148-9202	9168-9222				
*trn*S (AGN)	9255-9308	9220-9273	9184-9260	9223-9275				
*nad*2	9320-10151	9252-10119	9269-10100	9288-10119	ATA/TAG (277)	ATA/TAA (289)	ATA/TAA (277)	ATA/TAG (277)
*trn*I	10156-10214	10125-10180	10105-10161	10124-10180				
*trn*R	10217-10272	10181-10236	10162-10216	10187-10242				
*trn*Q	10279-10334	10236-10292	10219-10273	10249-10303				
*trn*F	10345-10402	10298-10356	10279-10335	10312-10367				
*cob*	10402-11512	10353-11466	10333-11446	10368-11478	ATT/TAA (370)	ATG/TAG (371)	ATA/TAA (371)	ATT/TAA (370)
*trn*L (CUN)	11514-11570	11468-11523	11448-11502	11480-11534				
*cox*3	11570-12338	11523-12291	11503-12271	11535-12303	ATT/TAA (256)	ATT/TAA (256)	ATT/TAA (256)	ATT/TAA (256)
*trn*T	12336-12392	12289-12346	12269-12324	12301-12355				
*nad*4	12420-13620	12376-13573	12286-13552	12380-13583	ATA/TAA (400)	ATG/TAA (399)	ATA/TAA (422)	ATA/TAA (400)

^1^OTU-C = *H. macropi* from *Macropus robustus robustus*; ^2^OTU-G = *H. macropi* from *Macropus bicolor*, ^3^OTU-J = *H. macropi* from *Thylogale billardierii*.

Twenty-two tRNA genes were predicted from each mt genome of the three OTUs of *H. macropi*, and these genes varied from 53–64 nucleotides in length. Their positions in the genome were the same for all OTUs and for *Ma. ocydromi* (Table 1). The two ribosomal RNA genes (*rrn*S and *rrn*L) were separated by *nad*3, *nad*5, *nad*6, *nad*4L and 5 *trn* genes (Figure 1). The lengths of these *rrn* genes were 700 bp and 959 bp, respectively, which is similar to those reported previously for various nematodes, including *Ancylostoma duodenale*, *Necator americanus*, *Onchocerca volvulus*, *Strongyloides stercoralis* and *Xiphinema americanum*[23,25-28].

The AT-content of the mt genomes of *H. macropi* was 70-78%. Within the Strongylida, AT-richness in the mt genome usually ranges from 76.6-77.2% [25,29]. The nucleotide composition of the entire mt genome is biased toward T, and C is least favoured (Table 2), which is similar to other nematodes including *Caenorhabditis elegans*, *Dirofilaria immitis* and *Onchocerca volvulus*[30-32]. The AT content of the *rrn* genes for *H. macropi* and *Ma. ocydromi* ranged from 75–77% (Table 2). Given the AT bias (70-78%) in the mt genome of *H. macropi*, there was considerable bias in codon usage. Consequently, ATR (methionine), ATY (isoleucine), TTR (leucine), and TTY (phenylalanine) were the most commonly used codons (6.6-7.2%, 6.4-7.6%, 7.2-18% and 12.7-13%, respectively) (Table 3).

**Table 2:** Nucleotide composition (%) for the entire or regions of the mitochondrial genomes determined herein

**Species**		**Length (bp)**	**A**	**C**	**T**	**G**	**A + T**
*H. macropi* (from *Macropus robustus robustus*)	Entire sequence	13928	28.0	7.2	45.6	17.6	73.6
	Protein genes	10520	25.5	7.4	46.9	18.5	72.4
	RNA genes	1689	36.1	6.6	41.2	14.5	77.3
*H. macropi* (from *Macropus bicolor*)	Entire sequence	13883	28.8	6.7	46.1	16.8	74.9
	Protein genes	10553	26.4	6.9	47.4	17.7	73.7
	RNA genes	1689	35.8	6.2	42.0	14.3	77.8
*H. macropi* (from *Thylogale billardierii*)	Entire sequence	13868	25.8	7.2	46.2	19.2	72.0
	Protein genes	10522	23.3	7.4	47.4	20.3	70.7
	RNA genes	1689	34.2	6.8	41.4	15.6	75.6
*Macropicola ocydromi*	Entire sequence	13743	28.9	6.6	45.7	17.2	74.6
	Protein genes	10480	26.5	6.8	47.0	18.0	73.5
	RNA genes	1689	36.0	6.2	41.3	14.9	77.3

**Table 3:** **Codon usages (%) in mitochondrial protein genes of *Hypodontus macropi *** **from three different hosts**

**Amino acid**	**Codon**	**Number of codons and percentage of codon usage (%)**			
		**OTU-C**^**1**^	**OTU-G**^**2**^	**OTU-J**^**3**^	***Ma. ocydromi***
**Non-polar**					
Alanine	GCN	101 (2.9)	103 (3.0)	99 (2.9)	96 (2.8)
Isoleucine	ATY	260 (7.5)	247 (7.1)	221 (6.4)	252 (7.3)
Leucine	CTN	361 (10.5)	14 (0.4)	30 (0.9)	12 (0.3)
Leucine	TTR	621 (18.0)	261 (7.1)	500 (14.5)	264 (7.7)
Methionine	ATR	233 (6.8)	245 (7.1)	226 (6.6)	248 (7.2)
Phenylalanine	TTY	447 (13.0)	438 (12.7)	450 (13.1)	438 (12.8)
Proline	CCN	80 (2.3)	81 (2.3)	81 (2.3)	81 (2.4)
Tryptophan	TGR	70 (2.0)	69 (2.0)	71 (2.1)	68 (2.0)
Valine	GTN	274 (8.0)	285 (8.2)	310 (9.0)	274 (8.0)
**Polar**					
Aspargine	AAY	141 (4.1)	140 (4.0)	130 (3.8)	140 (4.1)
Cysteine	TGY	45 (1.3)	45 (1.3)	51 (1.5)	47 (1.4)
Glutamine	CAR	43 (1.2)	42 (1.2)	40 (1.2)	42 (1.2)
Glycine	GGN	202 (5.9)	198 (5.7)	208 (6.0)	194 (5.7)
Serine	AGN	225 (6.5)	221 (6.4)	226 (6.6)	226 (6.6)
Serine	TCN	515 (15.0)	145 (4.2)	142 (4.1)	141 (4.1)
Threonine	ACN	119 (3.5)	113 (3.3)	116 (3.4)	117 (3.4)
Tyrosine	TAY	189 (5.5)	197 (5.7)	190 (5.5)	187 (5.4)
**Acidic**					
Aspartate	GAY	62 (1.8)	63 (1.8)	64 (1.9)	60 (1.7)
Glutamate	GAR	77 (2.2)	78 (2.3)	77 (2.2)	78 (2.3)
**Basic**					
Arginine	CGN	31 (0.9)	31 (0.9)	31 (0.9)	31 (0.9)
Histidine	CAY	57 (1.7)	57 (1.6)	56 (1.6)	56 (1.6)
Lysine	AAR	112 (3.2)	115 (3.3)	111 (3.2)	114 (3.3)

^1^OTU-C = *H. macropi* from *Macropus robustus robustus*; ^2^OTU-G = *H. macropi* from *Macropus bicolor*; ^3^OTU-J = *H. macropi* from *Thylogale billardierii.*

### Genetic differences and phylogenetic relationships

**Figure 2:**
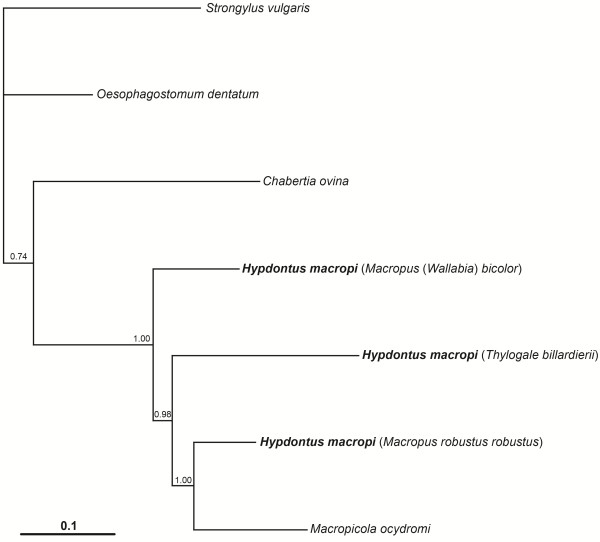
**Phylogenetic analysis of the concatenated amino acid sequences for protein coding genes of*Hypodontus macropi*.** Concatenated amino acid sequence data for all protein coding mitochondrial genes for three operational taxonomic units of *H. macropi*, from *Macropus robustus robustus*, *Macropus bicolor*, *Thylogale billardierii*, and from *Macropicola ocydromi* sequenced here as well as other concatenated sequence data representing complete mitochondrial genomes of *Chabertia ovina*, *Oesophagostomum dentatum* and *Strongylus vulgaris* (Strongyloidea) were analyzed using Bayesian Inference. The numbers above each tree branch represent the statistical support for each node (based on posterior probability score).

Pairwise comparisons of the amino acid (aa) sequences predicted for each of the 12 protein coding mt genes from OTU-C, OTU-G and OTU-J of *H. macropi* from the hosts *M. robustus*, *M. bicolor* and *T. billardierii*, respectively, are shown in Table 4. Most variation was detected in NAD6 between OTU-C and OTU-J (29%) and between OTU-G and OTU-J (26.9%), and in NAD4L between OTU-C and OTU-G (15.4%); moderate variation was detected in NAD1, NAD2, NAD3, NAD4 and NAD5 (mean: 9.8%) among the OTUs; least variation was detected in COX1, COX2 and COX3 (1.7-4.3%). Pairwise comparisons of concatenated amino acid sequences revealed sequence differences of 5.8% (OTU-C *versus* OTU-G), 18% (OTU-C *versus* OTU-J) and 10.0% (OTU-G *versus* OTU-J) (Table 4). These percentages are substantially higher than those detected within species of strongyloid nematodes [17,18,23]. Pairwise comparisons of the concatenated amino acid sequence of *Ma. ocydromi* with those of OTU-C, OTU-G and OTU-J revealed sequence differences of 30.4%, 15.9% and 31.0%, respectively (Table 5). The phylogenetic analysis of the aa sequence data showed that all three OTUs of *H. macropi* and *Ma. ocydromi* formed a distinct group, with maximum statistical support (posterior probability [pp] = 1.00), to the exclusion of the other strongyloid nematodes included here (i.e., *Oesophagostomum dentatum*, *Chabertia ovina* and *Strongylus vulgaris*) (Figure 2). In this analysis, OTU-C from *M. r. robustus* grouped together with *Ma. ocydromi* (pp = 1.00) to the exclusion of OTU-G (*M. bicolor*) and OTU-J (*T. billardierii*). While the three OTUs of *H. macropi* did form a group with *C. ovina*, statistical support for this grouping was moderate (pp = 0.74) (Figure 2).

**Table 4:** Pairwise comparison of the amino acid sequences of the 12 protein coding mitochondrial genes

**Protein**	**OTU-G vs. OTU-C**	**OTU-J vs. OTU-C**	**OTU-G vs. OTU-J**
ATP6	4.5	13.6	15.7
COB	5.4	6.2	9.7
COX1	2.0	4.0	3.2
COX2	1.7	4.3	3.9
COX3	3.1	4.3	3.9
NAD1	3.0	6.4	6.7
NAD2	13.2	16.7	16.6
NAD3	5.4	6.3	7.15
NAD4	7.4	11.8	11.7
NAD4L	15.4	14.1	9.0
NAD5	8.1	13.5	13.5
NAD6	5.6	29.0	26.9
All genes	5.8	18.0	10.0

These percentage differences are based on comparisons between three operational taxonomic units (OTUs) of *Hypodontus macropi* from three different hosts (i.e., OTU-C from *Macropus robustus robustus*; OTU-G from *Macropus bicolor*, OTU-J from *Thylogale billardierii*).

**Table 5:** Pairwise comparison of the amino acid sequences of the 12 protein coding mitochondrial genes

**Protein**	**OTU-C vs. Mo**	**OTU-G vs. Mo**	**OTU-J vs. Mo**
ATP6	5.5	5.0	14.1
COB	5.1	7.8	9.7
COX1	2.9	2.1	3.6
COX2	1.7	1.7	3.9
COX3	2.7	3.1	5.9
NAD1	1.9	2.6	5.6
NAD2	10.3	13.5	16.4
NAD3	4.5	5.4	7.2
NAD4	8.9	13.9	14.6
NAD4L	9.0	11.5	10.3
NAD5	11.2	12.3	16.1
NAD6	30.4	15.9	31.0

These percentage differences are based on comparisons between three operational taxonomic units of *Hypodontus macropi* from three different hosts (i.e., OTU-C from *Macropus robustus robustus*; OTU-G from *Macropus bicolor*, OTU-J from *Thylogale billardierii*) and *Macropicola ocydromi* (*Mo*) from *Macropus fuliginosus.*

### Sliding window analyses

**Figure 3:**
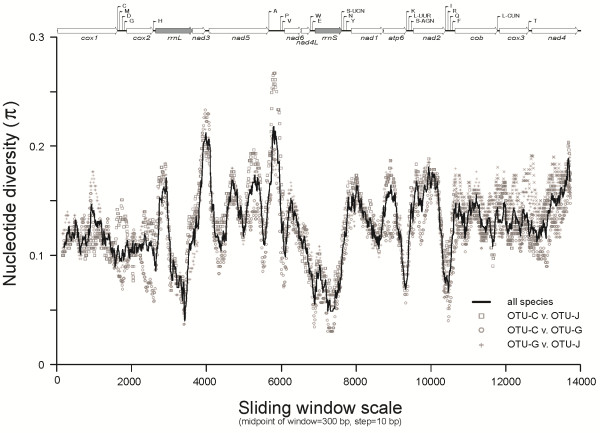
**Sliding window analysis of complete mt genome sequences of*Hypodontus macropi*from different hosts.** The three operational taxonomic units (OTUs) of *H. macropi* were from *Macropus robustus robustus* (OTU-C), *Macropus bicolor* (OTU-G) and *Thylogale billardierii* (OTU-J). The black line indicates nucleotide diversity comparing all the three OTUs; whereas three other symbols (a square, a circle and a plus sign) indicate pairwise comparisons across the mt genomes of OTU-C, OTU-G and OTU-J. Nucleotide diversity, measured iteratively every 10 bp over 300 bp windows of aligned mtDNA sequence data indicate peaks and troughs of sequence variability. Linearized maps of mtDNA are provided for each aligned data set, with the highest peaks of variability falling within the non-coding AT-rich regions.

Sliding window analyses were carried out to identify conserved and variable regions in mt genomes among all three OTUs. Results from these analyses are shown in Figure 3 for comparison across all the three OTUs and also for nucleotide diversities calculated from pairwise comparisons across the mt genomes of OTU-C, OTU-G and OTU-J. A nucleotide diversity pattern was broadly similar across the sliding window analyses and diversity within and between genes was relatively consistent across the genera. Greatest nucleotide diversity was detected within AT-rich (AT) region, followed by peaks of variation within NAD3, NAD4, NAD2 and NAD5. Gene-by-gene nucleotide diversity was highly variable, but, by far, the least variation was recorded within COX2. Overall, the full sliding window indicates a wealth of new genes capable of providing high levels of nucleotide variation for *H. macropi* population genetic studies.

## Discussion

Molecular tools offer an unprecedented opportunity to include new components in our studies on the discovery and description of parasite species. For example, molecular markers in the identification of parasite species can be used to (i) link morphologically indistinguishable life stages, such as larvae, to the adult stages of known species, (ii) elucidate life cycles by establishing the species that may serve as intermediate or paratenic hosts for larval stages of a parasite, and (iii) search for cryptic species [12]. However, Nadler and Pérez-Ponce de León [33] suggested that, in terms of explanatory power, single locus DNA barcodes and the barcoding-gap are insufficient approaches to delimit species, and concordance of independent information, including other genes, is required. In the present study, we sequenced the whole mt genomes of individuals of *H. macropi*, proposed to be distinct cryptic species based on MEE analysis and the ITS-2 sequences of specimens [8-11] from three different species of macropodid marsupial hosts. Based on the analysis of the complete mt genomes of three cryptic species, within the morphospecies *H. macropi*, the present study provides independent support that specimens of *H. macropi* from three species of macropodid hosts represent three genetically distinct species. The present mt genomic results support previous findings [8-11] from MEE and nuclear DNA-based analyses and indicate that *H. macropi* is represented by a complex of at least ten sibling species, one in each of *M. agilis, M. bicolor, M. dorsalis*, *M. rufogriseus*, *P. persephone*, *T. billardierii* and *T. stigmatica*, with the remaining three species occurring in *M. r. robustus* and *M. rufus*.

Although the ITS-2 rDNA has been shown to be a reliable genetic marker to distinguish among a variety of strongyloid nematodes [13-15] and this marker has been useful for discovering sibling species within various cloacinine nematodes in macropodid marsupials [34-38], hypotheses regarding such species need to be independently tested, preferably with sequence data from additional loci [33]. For this purpose, we chose mtDNA genes because of their relatively rapid rate of substitution, coupled to the smaller effective population size of mtDNA and the provision of evidence of lineage exclusivity in a shorter period of time (on average) following speciation [39,40]. Using mt datasets, based upon pairwise comparisons of concatenated amino acid sequences inferred herein, we found a wide range of sequence differences of among different OTUs (5.8 – 18%) (see Table 4), and this magnitude is consistent with the previous findings of Chilton et al. [11] using the ITS-2 rDNA. These authors also found a wide variation in the ITS-2 sequences among three OTUs i.e., genotype (G) 8 (OTU-G, *M. bicolor*), G13 (OTU-C, *M. r. robustus*), and G36 (OTU-J, *T. billardierii*) of *H. macropi*. Based on the ITS-2 sequences, the highest sequence variation detected was between OTU-C [GenBank: HE866725] and OTU-G [GenBank: HE866720] (33.3%), followed by OTU-C [GenBank: HE866725] and OTU-J [GenBank: HE866748] (27.8%) and OTU-G [GenBank: HE866720] and OTU-J [GenBank: HE866748] (25.8%).

In nematodes, mt DNA is usually more variable in sequence within a species than the ITS-2 and other rDNA regions [14], indicating that mt gene regions are well suited for studying the population genetics of parasitic nematodes [14,17,41]. The sliding window analysis conducted herein displayed distinct patterns of nucleotide diversity among the three mt genomes representing OTUs-C, -G and -J of *H. macropi* (see Figure 3). In the present study, COX2 was revealed to be the gene with the lowest nucleotide diversity among these three OTUs*,* whereas NAD2, NAD3, NAD5 and NAD6 showed higher nucleotide diversity, which can be targeted to design primers for studying the population genetics of *H. macropi*. Given that cryptic species have been detected within the morphospecies *H. macropi*[8-11], low sequence variability is useful for the design of oligonucleotide primers that flank mt regions with high variability. Utilizing such primer sets, PCR-based SSCP analysis [42] could be applied to screen large numbers of individual specimens representing different host species and populations for cryptic species. Previous studies [10,11] have shown the merit of SSCP for exploring the genetic variation in various populations of *H. macropi*, and this approach could be applied to large-scale studies of *H. macropi* specimens representing distinct OTUs. It is hoped that major advances in the next-generation sequencing technology (e.g., Illumina) [43] will allow selective whole genome sequencing of cryptic species of nematodes, thereby helping to re-classify them by employing detailed phylogenetic and comparative genetic analyses.

## Conclusions

The present analyses of mt proteomic sequence datasets clearly supported the hypothesis that *H. macropi* from *M. robustus robustus, M. bicolor* and *T. billardierii* represent distinct species. This study emphasizes the importance and utility of the mt genomic datasets for parasites from macropodid marsupials as a basis for systematic, ecological and biological studies, and the specific diagnosis of infections. In particular, studying cryptic species within the morphospecies *H. macropi* provides a stimulus to re-explore the relationships of sibling species using complete mt genomic and proteomic sequence data sets. Although the present study focused on *H. macropi*, the approach employed here has major implications for the study of a wide range of invertebrates and vertebrates.

## Methods

### Collection of nematodes and isolation of genomic DNA

Adult *H. macropi* individuals were collected opportunistically from the intestines of *M. r. robustus*, *M. bicolor* and *T. billardierii*. The nematodes from these three host species were designated as operational taxonomic units (OTUs), namely OTU-C, OTU-G and OTU-J, respectively, according to Chilton et al. [8]. A specimen of a related species, *Macropicola ocydromi*, was also collected from *Macropus fuliginosus* and included for genetic comparison with OTUs of *H. macropi*. Individual worms were stored (in individual tubes) at −80°C until required. Upon thawing, individual nematodes were washed extensively in physiological saline, pH 7.4. The anterior and posterior ends of each nematode were excised, cleared in lactophenol for identification and then stored in 70% ethanol and retained as voucher specimens, which have been deposited in the South Australian Museum (SAM 46024, 46038 and 46053; see Chilton et al. [11]), Adelaide, Australia. The remainder of each worm was used for the extraction of genomic DNA using a small-scale sodium dodecyl-sulphate (SDS)/proteinase K digestion and column-purification (Wizard DNA Clean-Up Kit, Promega, USA) [42]. The identity of each specimen was verified by PCR-based amplification of the ITS-2 using an established method, followed by mini-column purification (Wizard PCR-Preps, Promega) of the amplicon and subsequent automated (bidirectional) sequencing (BigDye chemistry v.3.1) [42].

### Sequencing and assembly of mt genomes

The complete mt genome of each worm was amplified by long-PCR (Advantage 2 kit; BD Biosciences) as two overlapping amplicons (“large” and “small”), using two (relatively) conserved primer sets, 5 F-40R and 39 F-42R, respectively [26,44] and with appropriate positive and negative (i.e., no template) controls. The cycling conditions used were 95°C for 1 min (initial denaturation); then 95°C for 15 s (denaturation), 50°C (for primer set 39 F-42R) or 55°C (for primer set 5 F-40R) for 15 s (annealing), and 60°C for 3 min (for primer set 39 F-42R) or 68°C for 6 min (for primer set 5 F-40R) (extension) for 35 cycles, and a final extension at 60°C for 3 min (for primer set 39 F-42R) or 68°C for 6 min (for primer set 5 F-40R). Products were consistently produced from the positive control samples (*Ascaris suum*); in no case was a product detected for any of the negative controls. Products were detected in 1% (w/v) agarose gels after ethidium-bromide staining and ultraviolet transillumination, and were then purified over a mini-column (Wizard, Promega) and quantified spectrophotometrically (ND-1000 UV–VIS spectrophotometer v.3.2.1; NanoDrop Technologies). Following electrophoretic verification of quality, the two amplicons (~5 kb and ~10 kb; 2.5 μg of each), spanning the mt genome of each worm specimen, were pooled and subsequently sequenced using the 454 Genome Sequencer FLX (Roche) [45]. The consensus mt genome sequences [GenBank: KF361317-KF361320] were each assembled automatically, using the CAP3 program [46], from individual reads (of ~300 bp).

### Bioinformatic pipeline for mt genomic annotation and analysis of sequence data

Following assembly, the genes and features of each mt genome from each worm were annotated using a semi-automated pipeline [21]. In brief, each protein coding mt gene was identified by local alignment comparison (performed in all six reading frames) using amino acid sequences predicted (employing invertebrate mitochondrial code) from the mt genome of a reference species (i.e., *Oesophagostomum dentatum* [GenBank: NC_013817]. The large and small ribosomal RNA subunit genes (*rrn*S and *rrn*L) were identified by local alignment (i.e., using nucleotide sequence data) using the same approach. All transfer RNA (tRNA) genes were detected and identified in a three-step process. Initially, all possible tRNA genes present in each consensus sequence were predicted (from both strands) based on a folding structure, using scalable models based on the standard nematode mt tRNAs [18]. All predicted tRNA genes were clustered into groups based on their anti-codon sequence and provisionally identified based on the amino acid encoded by this anti-codon. Two separate tRNA gene groups were predicted each for leucine (one each for the anticodons CUN and UUR, respectively) and for serine (one each for the anticodons AGN and UCN, respectively), as these tRNA genes have been shown to be duplicated in most invertebrate mt genomes, including those of nematodes [18]. All predicted tRNAs within each amino acid group were ranked based on structural “strength” (as inferred by the number of mismatched nt pairs in each stem), and the 100 best-scoring structures for each group were compared by BLASTn alignment against a database comprising all tRNA gene sequences for each amino acid of all published nematode mt genome sequences (available *via*http://drake.physics.mcmaster.ca/ogre/; [47]). All tRNA genes of each mt genome were then identified and annotated based on having the highest sequence identity to known nematode tRNA genes. Annotated sequence data were imported into the program SEQUIN (available *via*http://www.ncbi.nlm.nih.gov/Sequin/) for final verification of the mt genomic structure and subsequent, direct submission to the GenBank database.

### Alignment and phylogenetic analysis of concatenated nucleotide or amino acid sequence data

The amino acid sequences were conceptually translated from the mt genomes of the three individuals of *H. macropi* and the specimen of *Ma. ocydromi*, and then concatenated separately. The amino acid sequences derived from published mt genomes from nematodes [Strongylida: *Chabertia ovina* [GenBank: NC_013831], *Oesophagostomum dentatum* [GenBank: NC_013817] and *Strongylus vulgaris* [GenBank: NC_013818] [see [21]] were also used for comparative purposes. These amino acid sequences (considering all homologous characters) were aligned using MUSCLE [48]. The amino acid sequences of *H. macropi* and *Ma. ocydromi* were then aligned using BioEdit [49] and subjected to phylogenetic analysis using Bayesian inference (BI), employing the Markov chain Monte Carlo (MCMC) method in MrBayes 3.1.2 [50,51]. The likelihood parameters for BI were based on the Akaike Information Criteria (AIC) test in Prottest v2.4 [52]. The “best” model for the amino acid sequences was a fixed-rate Poisson model [53]. Estimates of the base frequencies, the substitution rate model matrix and the proportion of invariable sites were fixed. Posterior probabilities (pp) were calculated using 2,000,000 generations, employing four simultaneous tree-building chains, with every 100th tree being saved.

### Sliding window analysis

This analysis was performed on the aligned, complete mt genome sequences of the three OTUs of *H. macropi* using DnaSP v.5 [54]. The alignment of these sequences was achieved using MUSCLE v.3.8 [47], as implemented in SeaView v.4 [55]. Keeping the nucleotides in frame, there were no ambiguously aligned regions. A sliding window of 300 bp (steps of 10 bp) was used to estimate nucleotide diversity (π) between the three OTUs of *H. macropi*; indels were excluded using DnaSP. Nucleotide diversity for the entire alignments was plotted against midpoint positions of each window, and gene boundaries were defined.

## Abbreviations

atp6: Adenosine triphosphatase subunit 6; cox: Cytochrome *c* subunit; cytb: Cytochrome *b* subunit; ITS-2: Second internal transcribed spacer; Mt: Mitochondrial; MEE: Multilocus enzyme electrophoresis; nad: Nicotinamide dehydrogenase subunit; OTU: Operational taxonomic unit; ORF: Open reading frame; rDNA: Nuclear ribosomal DNA; rrnL: Ribosomal large subunit; rrnS: Ribosomal small subunit; SSCP: Single-strand conformation polymorphism; tRNA: Transfer RNA.

## Competing interests

The authors declare that they have no competing interests.

## Authors’ contributions

RBG, ARJ & TL conceived the project and attracted the funding; AJ carried out molecular laboratory work; NM assisted with the bioinformatics analysis; TL performed the sliding window analysis; AJ, IB, NBC, ARJ, TL, & RBG carried out data analysis and interpretation; RBG, AJ & IB wrote the draft manuscript. All authors read and approved the final version of the manuscript.
